# Genome-wide identification and expression analysis of the *VQ* gene family in *Cucurbita pepo* L.

**DOI:** 10.7717/peerj.12827

**Published:** 2022-01-21

**Authors:** Ke Xu, Ping Wang

**Affiliations:** College of Horticulture and Plant Protection, Inner Mongolia Agricultural University, Huhehaote, Inner Mongolia, China

**Keywords:** VQ genes family, Expression pattern, Powdery mildew, *Cucurbita pepo* L.

## Abstract

VQ protein is a plant specific protein, which plays an important role in plant growth and development and biological and abiotic stress response. This study aimed to systematically analyze for the first time the *VQ* of *Cucurbita pepo* and understand their expression patterns in response to different stimuli. Herein, 44 *VQ* genes were identified, which were divided into eight groups (I–VIII) based on phylogenetic analysis. Two genes (*CpVQ1* and *CpVQ2*) could not be located on the chromosome, whereas the remaining *CpVQ* genes were randomly distributed on the chromosomes, except for chromosomes 15 and 18. Noteworthy, the main event driving the expansion of the *VQ* gene family was chromosome fragment duplication. Based on qRT-PCR analysis, *VQ* genes are expressed in different tissues, and *VQ* genes are differentially regulated under a variety of abiotic stresses and powdery mildew stress, indicating that they play an important role in plant stress response and other aspects. This report presents the first systematic analysis of *VQ* genes from *C. pepo* and provides a solid foundation for further research of the specific functions of VQ proteins.

## Introduction

VQ protein is a highly conserved transcription factor that is widely expressed in biology, including several monocotyledonous and dicotyledonous plants (Jing & Lin, 2015; Song et al., 2016; Ding et al., 2019). This protein owns its name to its VQ motifs, which allows it to interact with WRKY transcription factors to regulate plant growth and development (Lai et al., 2011). For example, *VQ10* interacts with *WRKY25* and *WRKY33*, which when simultaneously overexpressed can impair plant growth (Cheng et al., 2012). In *Arabidopsis*, *AtWRKY2/34* and *AtVQ20* form a complex that negatively regulates the expression of MYB transcription factors to regulate the development and function of pollen (Lei et al., 2017; Lei, Ma & Yu, 2018). The *ATVQ8* gene affects leaf development, making the leaves appear yellow-green throughout the growth period, causing plant malnutrition and affecting the normal growth of *Arabidopsis* plants. Plants overexpressing *VQ17*, *VQ18*, and *VQ22* have slow growth and yellow leaves, and the leaf area becomes smaller (Cheng et al., 2012). Moreover, *IKU1* encodes a protein containing a VQ motif that is involved in the regulation of endosperm development, which in turn affects seed size (Wang et al., 2010a).

VQ plays an important protective role on plants, allowing them to fight pathogenic bacteria and other biological and abiotic stresses (Jiang, Sevugan & Ramachandran, 2018). For example, *OsVQ13* is highly expressed at 6 h after bacterial blight infection; thus, it may be involved in the resistance pathway of *Oryza sativa* to bacterial blight (Kim et al., 2013a). Some *PbrVQ* genes are differentially expressed under gibberellin and black spot stress (Cao et al., 2018). Furthermore, transgenic *Arabidopsis* overexpressing *AtVQ15* is highly sensitive to salt stress during seed germination and seedling growth, whereas mutants of this gene show strong salt tolerance (Perruc et al., 2004). In response to harsh environments, plants have evolved several complex mechanisms to deal with external stimuli (Fujita et al., 2006). This response is caused by the interaction of transcription factors and cofactors to coordinate the transcription mechanisms of the plant in response to the surrounding environment (Wray et al., 2003). For example, *AtVQ9* acts antagonistically with *AtWRKY8* to mediate responses to salt stress (Hu et al., 2013). Studies have shown that *AtVQ10* and *WRKY8* form a complex in the nucleus and positively regulate the resistance of *Arabidopsis* to *Botrytis cinerea* (Chen et al., 2018). The banana fruit *MaWRKY26* transcription factor physically interacts with the VQ motif-containing protein *MaVQ5*, which leads to attenuated *MaWRKY26*-induced transactivation of jasmonic acid biosynthetic genes that are associated with various stress responses (Ye et al., 2016).

*C. pepo* belongs to the *Cucurbita* genus of the Cucurbitaceae family. Because it contains several nutrients, it has high edible and medicinal value, and is widely cultivated worldwide. During the cultivation process, *C. pepo* is susceptible to various biological and abiotic stresses that will affect the yield and quality of the fruit. Therefore, identification of resistance genes has great significance for improving the yield and quality of *C. pepo* through molecular breeding. Currently, 34, 39, 75, and 61 *VQ* genes have been identified in *Arabidopsis*, *O. sativa*, *Glycine max* and Maize, respectively (Cheng et al., 2012; Kim et al., 2013a; Wang et al., 2019; Song et al., 2016). However, there is no detailed description of the *VQ* genes of *C. pepo*. In this study, we investigated the *VQ* genes in the *C. pepo* genome, and analyzed their phylogeny, gene structure, chromosomal location, and collinearity for a comprehensive analysis. In addition, we also analyzed the expression levels of *CpVQs* under different abiotic stresses and powdery mildew stress. This study is expected to provide basic information for the identification and classification of *CpVQ* genes. Further experimental analysis can allow us to understand the functions of *CpVQ*s involved in plant stress response.

## Materials and Methods

### Identification of *VQ* genes in *C. pepo*

In present study, two methods were used to identify complete VQ members in *C. pepo*. Firstly, The Hidden Markov Model (HMM) profiles of the VQ motif (PF05678) was downloaded from PFAM protein family database (http://pfam.xfam.org/) (Punta et al., 2004). Then the model as a probe to perform a BLASTp against the *C. pepo* genome database using HMMER 3.0. The cut-off E-value was set as 0.1. Secondly, The known VQ motif-containing members of *A. thaliana* were obtained from The *Arabidopsis* Information Resource (TAIR) and database (http://arabidopsis.org) were used as queries to conduct a local BLASTp against the protein database of *C. pepo*. The targeting genes with similarity of E-value less than 1e^–20^ were retained for the further analysis. We set e as two values of 0.1 and 1e^–20^ for filtering. We set E-value to 0.1 for the initial filtering, and then set E-value to 1e^–20^ for in-depth filtering. After merging the two results, the candidate *VQ* genes were evaluated using the online program PFAM (http://pfam.xfam.org/search), Conserved Domains DataBase Search (https://www.ncbi.nlm.nih.gov/Structure/cdd/wrpsb.cgi) and SMART tool (http://smart.embl-heidelberg.de/) to confirm the presence of VQ motif. The biophysical properties of VQ members, such as, peptide length, molecular weight (MW), isoelectric point (pI) were predicted using the online ExPasy program (http://www.expasy.org/tools/) (Wilkins et al., 1999).

### Multiple sequence alignment and phylogenetic analysis

In order to investigate the evolutionary relationships and classification of VQ members in *C. pepo*, we firstly conducted a multiple sequence alignment based on the full-length amino acid sequence of all *CpVQs* using the ClustalW tool with auto strategy parameters. According to the alignment results, we constructed a phylogenetic tree using the neighbor-joining (NJ) method with MEGAX software (Tamura et al., 2011). Branch support for the tree topology was estimated by using a bootstrap analysis with 1,000 replicates. The phylogenetic tree was illustrated using the online ITOL (http://itol.embl.de/help.cgi) online tool.

### Gene structure analysis and conserved motif detection

The online program Gene Structure Display Server (GSDS) (http://gsds.cbi.pku.edu.cn/) was used to draw a diagrammatic sketch of the intron-exon structure by comparing their coding sequence (CDSs) with their corresponding genomic DNA sequences. The conserved motifs of *CpVQs* were detected by the online tool MEME (http://meme.ebi.edu.au/). The parameters were set as follows: zero or one occurrence per sequence; maximum number of motifs: 20; and other optional parameters was set default (Bailey et al., 2015).

### Chromosome locations, gene duplication and collinearity analysis

For the further investigation of evolution in *CpVQs*, we firstly constructed the distribution map of VQ members in *C. pepo*. The physical positions of all *VQ* genes were retrieved from the GFF3 annotation file using a local script and the schematic diagram of chromosomal location was visualized by TBTOOLS (Chen et al., 2020a). For the identification of tandem duplication events and segmental duplication events in CpVQ family, all *CpVQs* amino acid sequences were aligned using BLASTp, with an e-value of 1e^–10^. As previous research described, two or more genes located on the same chromosome were arranged in a 200 kb distance and shared more than 70% identity as analyzed with BLASTp can be defined as tandem duplication events. The segmental duplication events were identified by using the Multiple collinear scanning toolkits (MCScanX) with default parameters (Wang et al., 2012a). The Circos program was used to draw collinearity maps to exhibit duplicated gene pairs between *CpVQ* genes as well as the synteny blocks of the orthologous *VQ* genes between *C. pepo* and *Arabidopsis thaliana*, *Cucumis melo*, *Oryza sativa*, *Zea mays*.

### Calculating Ka and Ks

The Ka and Ks were calculated to assess the selection history and divergence time of gene families. The values of synonymous (Ks) and nonsynonymous (Ka) substitutions of duplicated *VQ* genes were estimated by using the KaKs_Calculator 2.0 with the NG method (Wang et al., 2010b). The divergence time (T) was calculated using the formula T = Ks/(2 × 6.1 × 10^−9^) × 10^−6^ million years ago (MYA) (Kim et al., 2013a, 2013b).

### *VQ* gene expression analysis of *C. pepo*

The transcriptome expression data of *VQ* genes under *Podosphaera xanthii* stress was available from our laboratory. The transcript abundance was represented by fragments per kilobase of exon per million mapped reads (FPKM) values which were calculated based on RNA-Seq reads. The heatmaps showing expression profiles were generated using log10-transformed FPKM values. The results were presented as heatmaps using TBTOOLS software (Chen et al., 2020a).

### Plant materials and treatments

*Cucurbita pepo* (Self-delivered F2) was used in present study. Seeds were planted in a 1:1 (w/w) mixture of soil and sand, cultured in an artificial climatic chamber kept at 30/22 °C with a 18/6 h photoperiod (day/night). Seedlings that germinated after 8 weeks were subjected to different stress conditions: 200 mM NaCl solution, 20% PEG6000 (drought), 4 °C (cold), immersing (waterlogging) and cultured for a total of 24 h, leaves were collected after 0, 12 and 24 h. In order to analyze the expression of *VQ* genes in different tissues, we collected plant roots, stems, and leaves for RNA preparation. All of the samples were immediately frozen in liquid nitrogen and stored at −80 °C for subsequent total RNA extraction. All samples were tested with three technical replicates and three independent biological replicates.

### RNA extraction and quantitative real-time PCR (qRT-PCR)

Total RNA was extracted from *C. peop* using RNAsimple Total RNA Kit (TIANGEN BIOTECH, Beijing, China) according to the manufacturer’s instructions. First-strand cDNA synthesis was accomplished using TransScript One-Step gDNA Removal and cDNA Synthesis SuperMix (Transgen Biotech, Beijing, China). Quantitative Real-time PCR (qRT-PCR) was performed using TB Green *Premix Ex Taq* II (TliRNaseH Plus) (RR420Q TaKaRa Biotechnology, Beijing, China) on an FTC-3000P system (Funglyn Biotech, Toronto, Canada) with the primers listed in Table S1. The reaction procedure was completed under the following program: 30 s of pre-denaturation at 95 °C, 40 cycles of 5 s at 95 °C, and 30 s at 60 °C, and 4 °C to finish. All samples were tested with three technical replicates and three independent biological replicates. The relative expression level was calculated while using the 2^−∆∆CT^ method (Livak & Schmittgen, 2001). The *actin* and *CAC* genes were used as internal control (Obrero et al., 2011).

## Results

### Identification and sequence analysis of *VQ* genes in *C. pepo*

After manually removing redundant entries through screening (manually delete the genes without VQ motif) and validation of the search results, a total of 44 *VQ* genes were identified within the whole genome of *C. pepo*, which were named *CpVQ1–CpVQ44* based on their physical locations on the chromosomes. The characteristics concerning each gene, including gene number, length of coding sequence and amino acid sequence, MW and PI of the proteins, are summarized in Table 1. Subsequent sequence analysis of these 44 *CpVQ*s showed that the encoded CpVQ proteins ranged from 73 amino acids (aa) (*CpVQ34*) to 628 amino acids (*CpVQ14*) in length (average length: 219 amino acids). Similar to previous studies in *Arabidopsis* and *O. sativa*, most VQ proteins contained less than 300 amino acids. The calculated molecular weight and isoelectric points of these proteins varied from 8.34 kDa (*CpVQ34*) to 67.83 kDa (*CpVQ14*), and 4.68 (*CpVQ40*) to 10.67 (*CpVQ19*), respectively. The length of the coding sequences of this gene family was between 222 and 1,887 bp (average length: 660 bp).

**Table 1:** List of all *VQ* genes identified in *C. pepo*.

Gene name	Gene locus	Chromosome location	Length (aa)	pI	Molecular weight (Da)	Family group
CpVQ1	Cp4.1LG00g04520	16693604–16694523	160	5.44	17,313.57	III
CpVQ2	Cp4.1LG00g15310	42412302–42412658	118	9.83	13,171.89	II
CpVQ3	Cp4.1LG01g01160	LG01: 3157885–3158544	219	9.03	22,938.39	V
CpVQ4	Cp4.1LG01g09330	LG01: 4248892–4250868	389	6.8	41,553.79	V
CpVQ5	Cp4.1LG01g21070	LG01: 17809037–17809507	156	8.13	17,422.62	II
CpVQ6	Cp4.1LG02g02700	LG02: 3577192–3577794	200	6.91	21,690.73	V
CpVQ7	Cp4.1LG02g03510	LG02: 3102280–3103518	283	9.16	30,711.29	V
CpVQ8	Cp4.1LG02g05130	LG02: 2005092–2009226	118	9.83	13,178.19	II
CpVQ9	Cp4.1LG03g04140	LG03: 2346972–2347676	234	6.53	25,197.91	VI
CpVQ10	Cp4.1LG04g14070	LG04: 11310784–11311509	241	5.94	25,762.45	II
CpVQ11	Cp4.1LG05g10550	LG05: 7053658–7054299	213	6.51	22,984.1	II
CpVQ12	Cp4.1LG06g02740	LG06: 1556739–1557260	173	10.13	18,950.64	IV
CpVQ13	Cp4.1LG06g04260	LG06: 2472160–2473164	334	9.91	35,686.43	VII
CpVQ14	Cp4.1LG06g05110	LG06: 3011929–3017926	628	5.57	67,834.1	V
CpVQ15	Cp4.1LG06g05610	LG06: 3325430–3326068	113	9.62	12,485.03	V
CpVQ16	Cp4.1LG06g06820	LG06: 4263922–4264659	245	8.71	26,210.14	IV
CpVQ17	Cp4.1LG07g01470	LG07: 802433–803374	313	10.08	33,653.95	VII
CpVQ18	Cp4.1LG07g09800	LG07: 8797116–8797493	125	6.05	14,220.9	III
CpVQ19	Cp4.1LG08g09380	LG08: 7407091–7407423	110	10.67	12,069.01	II
CpVQ20	Cp4.1LG08g13390	LG08: 9672254–9672643	129	4.84	14,612.33	III
CpVQ21	Cp4.1LG08g13490	LG08: 9735143–9735811	222	5.92	23,933.64	VI
CpVQ22	Cp4.1LG09g08360	LG09: 7719957–7720586	209	9.64	22,441.4	IV
CpVQ23	Cp4.1LG10g01130	LG10: 3319719–3322117	425	8.71	45,497.21	V
CpVQ24	Cp4.1LG10g01930	LG10: 2886496–2886996	166	9.23	18,288.05	VIII
CpVQ25	Cp4.1LG10g04880	LG10: 1115416–1116003	195	9.05	21,186.84	IV
CpVQ26	Cp4.1LG10g06710	LG10: 166830–167354	174	6.28	18,860.78	VI
CpVQ27	Cp4.1LG10g11090	LG10: 7510307–7511283	164	5.72	17,629.89	III
CpVQ28	Cp4.1LG10g12290	LG10: 9255696–9256184	162	10.05	18,042.53	IV
CpVQ29	Cp4.1LG11g04560	LG11: 2540172–2540996	274	10.43	30,231.7	VII
CpVQ30	Cp4.1LG12g07320	LG12: 6924419–6924844	141	5.91	15,294.24	IV
CpVQ31	Cp4.1LG13g04200	LG13: 6294724–6297911	282	9.99	31,079.58	II
CpVQ32	Cp4.1LG14g01330	LG14: 3722302–3723387	425	8.71	45,497.21	V
CpVQ33	Cp4.1LG14g03000	LG14: 2622622–2623233	203	9.68	21,226.55	V
CpVQ34	Cp4.1LG14g03270	LG14: 2365261–2365482	73	9.15	8,344.47	I
CpVQ35	Cp4.1LG16g00810	LG16: 1567379–1568005	208	6.3	22,405.61	II
CpVQ36	Cp4.1LG17g08680	LG17: 5075487–5075909	140	7.84	14,816.74	IV
CpVQ37	Cp4.1LG19g00160	LG19: 110455–110994	179	10.04	19,803.85	IV
CpVQ38	Cp4.1LG19g02600	LG19: 2163376–2163876	166	4.93	17,768.9	III
CpVQ39	Cp4.1LG19g02610	LG19: 2160888–2161388	166	4.93	17,768.9	III
CpVQ40	Cp4.1LG19g06070	LG19: 7655240–7655563	107	4.68	12,049.37	I
CpVQ41	Cp4.1LG19g07250	LG19: 7111871–7112545	224	9.62	24,493.82	IV
CpVQ42	Cp4.1LG19g09780	LG19: 5873440–5875876	344	6.26	36,896.06	V
CpVQ43	Cp4.1LG19g10580	LG19: 5411418–5411909	163	9.66	17,828.64	VIII
CpVQ44	Cp4.1LG20g04410	LG20: 2519217″2520794	335	8.85	36,902.06	IV

### Phylogenetic analysis of *CpVQs*

**Figure 1:**
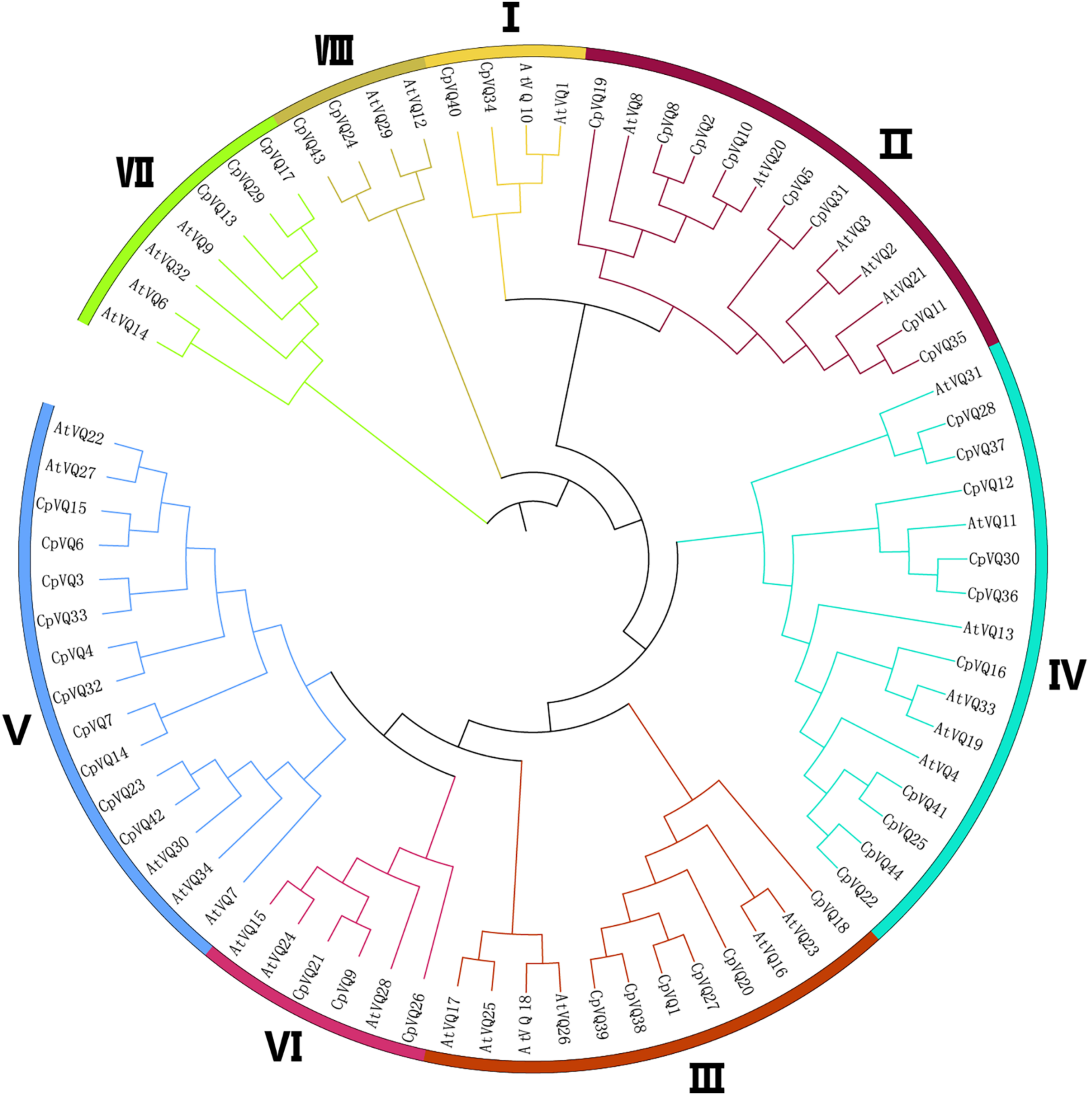
Phylogenetic tree analysis of the *VQ* genes in *C. pepo* *and Arabidopsis thaliana*. The clusters were designated as group I–VIII and indicated in a specific color.

To detect the evolutionary relationships and classification of the VQ family in *C. pepo*, unrooted phylogenetic Neighbor-Joining trees were constructed with the 44 CpVQ proteins and the known VQ protein from *Arabidopsis*. Through the analysis of the phylogenetic and structural features of the VQ domains, these proteins were divided into eight clades (I–VIII) based on the nomenclature of the *Arabidopsis VQs*, with two proteins in I and VIII groups, three each in VI and VII, six members in III, eight in II, and group IV and V have the biggest amount of proteins with 10 *VQs* (Fig. 1). When compared with the other groups, the size of group IV and V were significantly larger. These results were not completely consistent with previous studies in *A. thaliana*, *O. sativa*, and *Zea mays*. In addition, a phylogenic tree was built using the 44 CpVQ protein sequences, which indicated that the groups IV and V in *C. pepo* had more VQ members than those in *Arabidopsis*. In addition, it is also possible that the expansion of the two subgroups may have resulted from gene duplications.

### Gene structure and conserved motifs analysis

**Figure 2:**
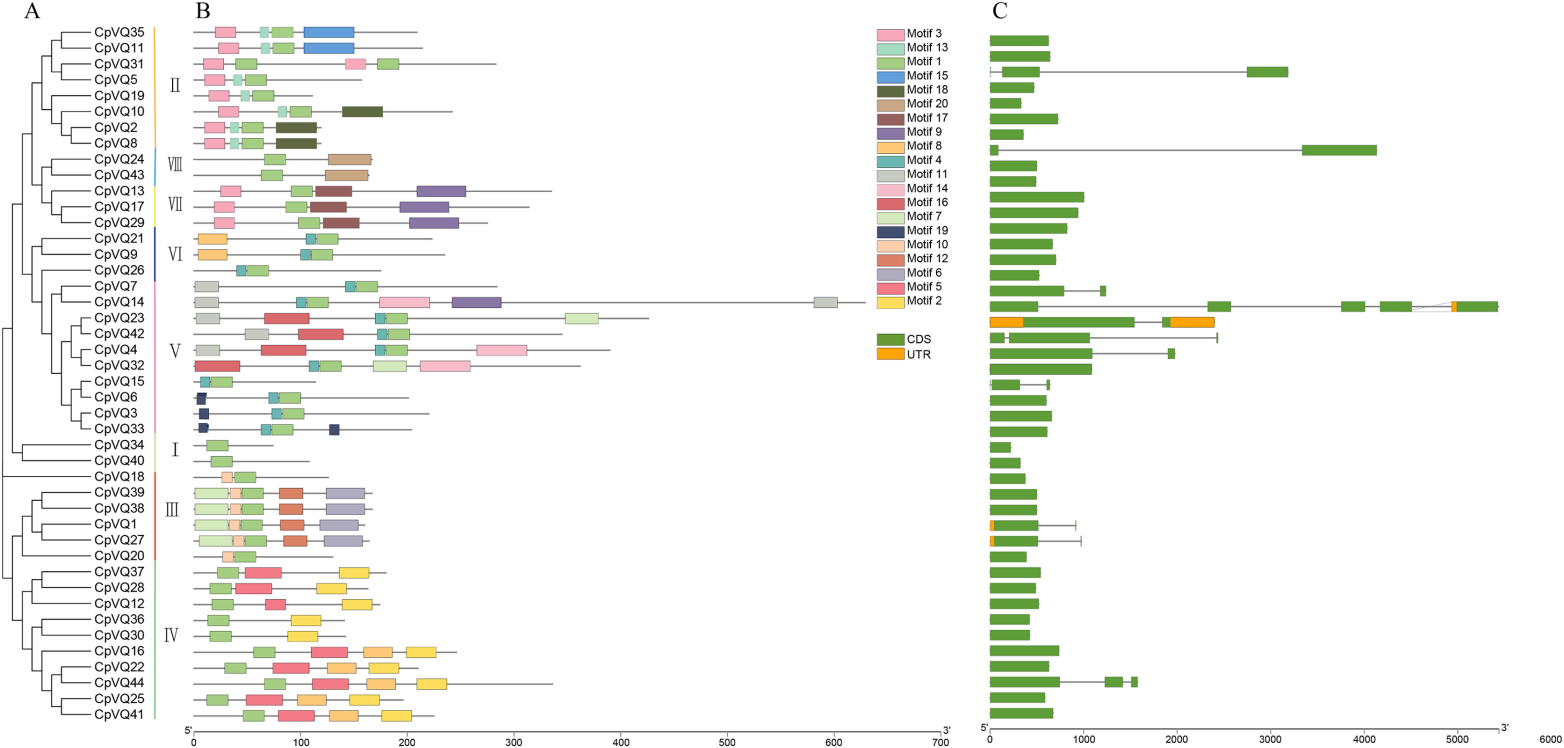
Phylogenetic tree, conserved motifs and gene structure in *CpVQs*. (A) Phylogenetic relationships. (B) Conserved motifs of the *CpVQs*. Each motif is represented by a number in colored box. (C) Exon/intron structures of *CpVQ* genes.

Gene structure can provide more information about the evolutionary relationship in a gene family; thus, the organization of exons and introns in *CpVQs* was investigated using the GSDS2.0 online tool. Structural analysis of *VQs* showed that half of the members of group V had introns, such as *CpVQ14* that had four introns, which may be related to their long sequences; genes in group VII have longer coding regions; whereas genes of group I have shorter coding regions than other groups. Moreover, 75% of *CpVQs* were found to be intronless genes, suggesting that many introns were lost during the long evolution. To further analyze the characteristics of *CpVQs*, the MEME online tool was used to predict the potential conserved motifs. A total of 20 motifs were identified, with lengths ranging between nine and 48 amino acids. Noteworthy, every *CpVQ* contained 1–6 conserved motifs (Fig. 2). Motif 1 contained the domain present in every VQ member; thus, representing an essential element in the VQ family. In addition, every group was found to have a clearly identifiable motif structure that distinguished it from the other groups. For example, Motif 2 and Motif 5 were prominent in Group IV, Motif 17 was only observed in Group VII, and Motif 6 was only present in Group III. It is worth mentioning that the gene structure and motif organization of *CpVQs* were similar within the same group, but diverged between groups, which also indicated that the phylogenetic classification was reliable.

### Chromosome mapping and duplication events analysis

**Figure 3:**
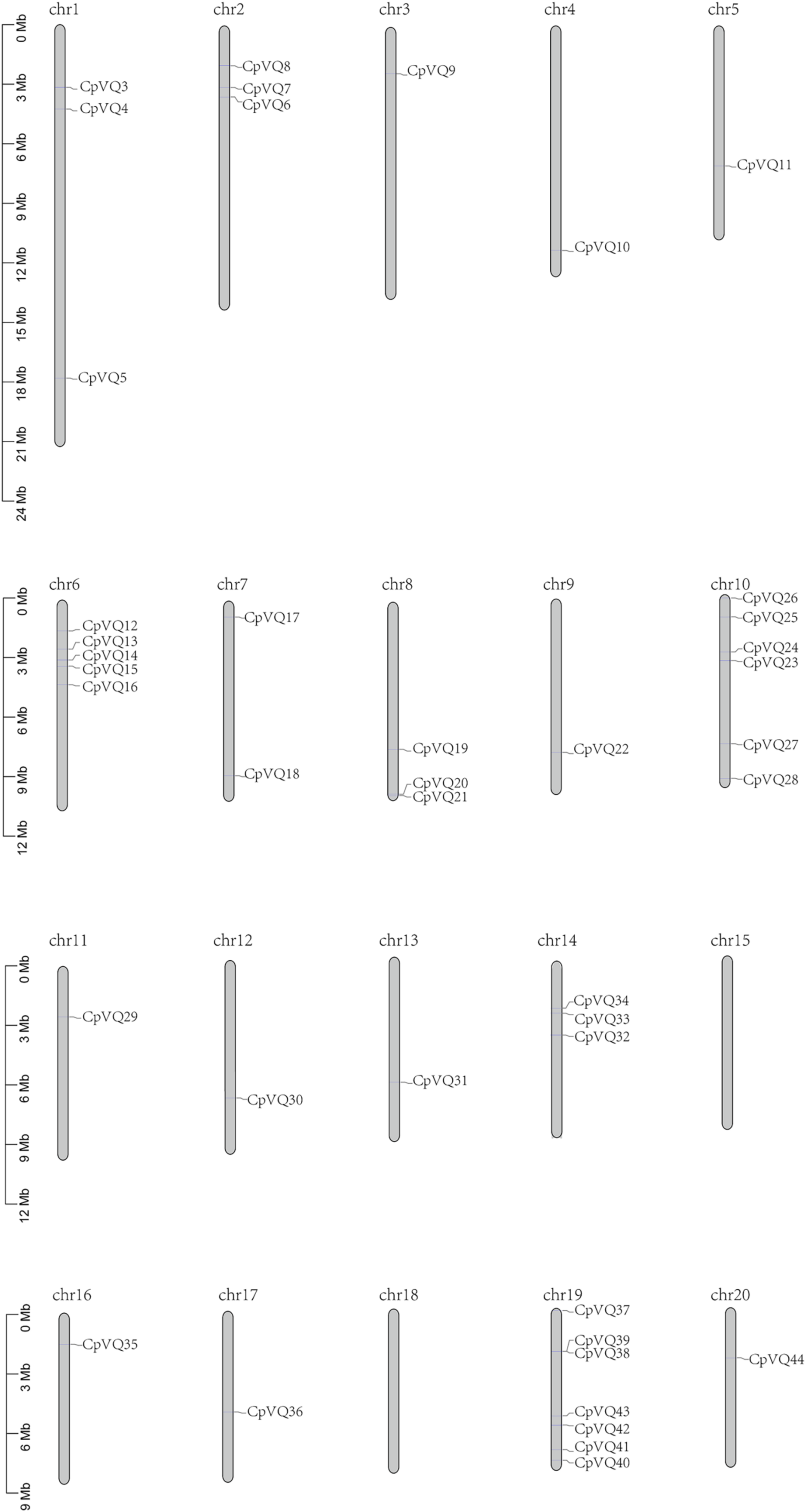
Chromosome location in *C. pepo*. Chromosome numbers were indicated above each chromosome. The size of a chromosome was indicated by its relative length.

Two *VQ* genes (*CpVQ1* and *CpVQ2*) could not be mapped on any chromosome, but the remaining *CpVQs* were randomly distributed on the chromosomes, except chomosomes 15 and 18 (Fig. 3). Chromosome 19 had the highest number of *CpVQs* (*n* = 7), followed by chromosome 10 (*n* = 6), chromosomes 1, 2, 6, 8, and 14 (*n* = 3), chromosome 7 (*n* = 2), and the remaining chromosomes contained one *VQ*. In addition, most *CpVQs* were found to be mainly located on the two ends of the chromosomes.

**Figure 4:**
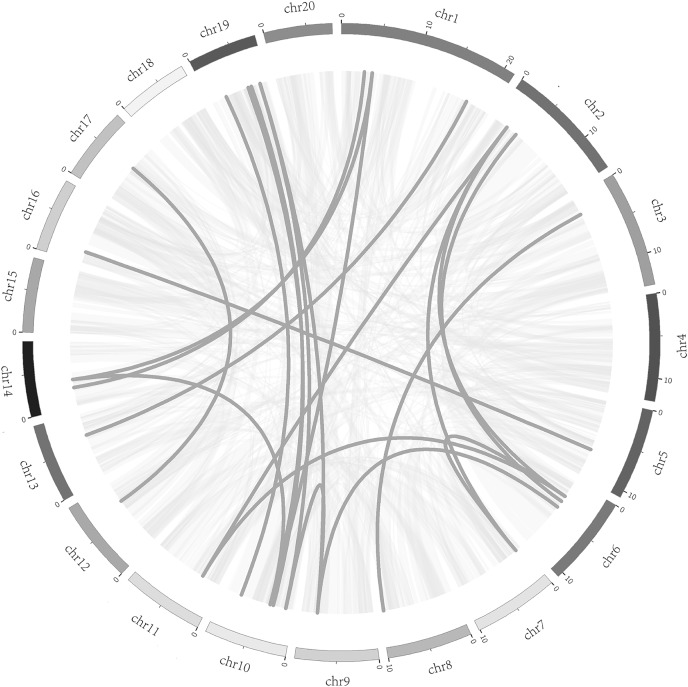
Syntenic analysis of *VQ* genes. Gray lines in the background indicate the collinear blocks within the *C. pepo* and oneself genomes, while the dark grey lines highlight the syntenic *VQ* gene pairs.

To better understand the evolution mechanism of *VQs* in plants, the tandem duplication and segmental duplication events of the CpVQ family were evaluated. Based on BLSTAp results, no tandem duplication event was detected. A total of 21 pairs with 28 *CpVQs* were identified in the whole genome (Fig. 4). Moreover, groups V and IV contained six and five segmental duplication events, respectively. Hence, these results suggested that segmental duplications were the primary driving force responsible for the expansion of the VQ family in *C. pepo*, which is consistent with previous reports.

### Synteny analysis of *VQ* genes

**Figure 5:**
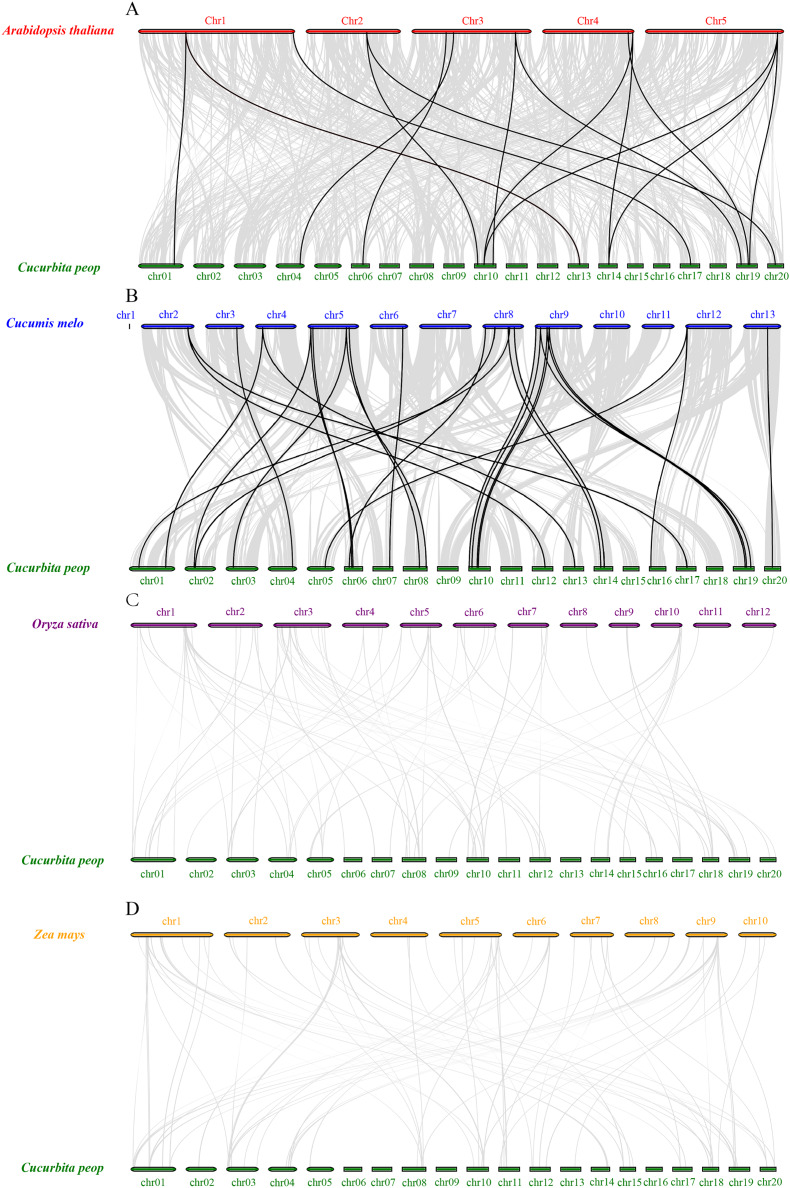
Synteny analysis of *VQ* genes between *C. pepo* and plant species. Synteny analysis of the *VQ* genes between (A) *C. pepo* and *Arabidopsis thaliana*; (B) *C. pepo* and *Cucumis melo*; (C) *C. pepo* and *Oryza sativa*; (D) *C. pepo* and *Zea mays*. Gray lines in the background indicate the collinear blocks within the *C. pepo* and other plant genomes, while the black lines highlight the syntenic *VQ* gene pairs.

To further understand the expansion mechanisms of the CpVQ family, we constructed comparative syntenic maps of *C. pepo* associated with four representative species, including two dicots (*Arabidopsis* and *Cucumis melo*) and two monocots (*O. sativa* and *Z. mays*). Overall, the *CpVQ* genes had the most homologous gene pairs with *C. melo* (*n* = 27), followed by *Arabidopsis* (*n* = 16). However, no homologous gene pairs were observed between *C*. *pepo* and *O. sativa* or *Z. mays* (Fig. 5). The syntenic analysis results further suggested that the *VQ* genes are highly conserved in dicotyledonous plants.

We calculated the Ka, Ks and Ka/Ks ratios of all para-homologous gene pairs (Table 2) to explore the evolutionary constraints of repeated *CpVQs*. The Ka/Ks values of most gene pairs were less than 1.0, which indicated that these gene pairs had undergone purification selection pressure. In addition, 21 pairs of genes varied from 31 to 216 MYA.

**Table 2:** Ka, Ks and Ka/Ks values calculated for homologous *VQ* gene pairs.

Gene1	Gene2	Ka	Ks	Ka/Ks ratio	Differentiation time
CpVQ3	CpVQ33	0.143408874	0.41714233	0.343788832	34.19199426
CpVQ4	CpVQ23	0.56179148	1.733083058	0.324157274	142.0559883
CpVQ4	CpVQ32	0.114789648	0.572645983	0.200454821	46.93819533
CpVQ5	CpVQ31	0.244725293	0.735263756	0.332840142	60.26752102
CpVQ6	CpVQ15	1.018237411	1.77385189	0.574026172	145.3976959
CpVQ8	CpVQ13	0.074887932	0.430075478	0.174127416	35.25208839
CpVQ8	CpVQ17	0.302766804	1.921171796	0.157594862	157.473098
CpVQ8	CpVQ29	0.319999352	1.905949455	0.167894983	156.2253652
CpVQ9	CpVQ21	0.895462518	NaN	NaN	#VALUE!
CpVQ11	CpVQ35	0.083226176	0.468993438	0.177457016	38.4420851
CpVQ13	CpVQ17	0.304326774	1.957821306	0.155441548	160.4771563
CpVQ13	CpVQ29	0.301640689	2.424748395	0.12440082	198.7498684
CpVQ16	CpVQ22	0.351584842	2.560617395	0.137304715	209.8866717
CpVQ22	CpVQ25	0.193161647	1.846190406	0.104627153	151.3270824
CpVQ22	CpVQ41	0.206138134	1.465932276	0.140619139	120.1583833
CpVQ23	CpVQ32	0.614828236	2.638504545	0.233021481	216.2708644
CpVQ23	CpVQ42	0.202547017	0.536664673	0.377418203	43.98890764
CpVQ24	CpVQ43	0.142828118	0.572200927	0.249611826	46.90171536
CpVQ25	CpVQ41	0.111174117	0.440146678	0.252584246	36.07759653
CpVQ27	CpVQ39	0.182897122	0.455285224	0.401719872	37.31846099
CpVQ30	CpVQ36	0.207558725	0.385917729	0.537831536	31.6326007

### Expression pattern of the *CpVQ* genes in different tissues

**Figure 6:**
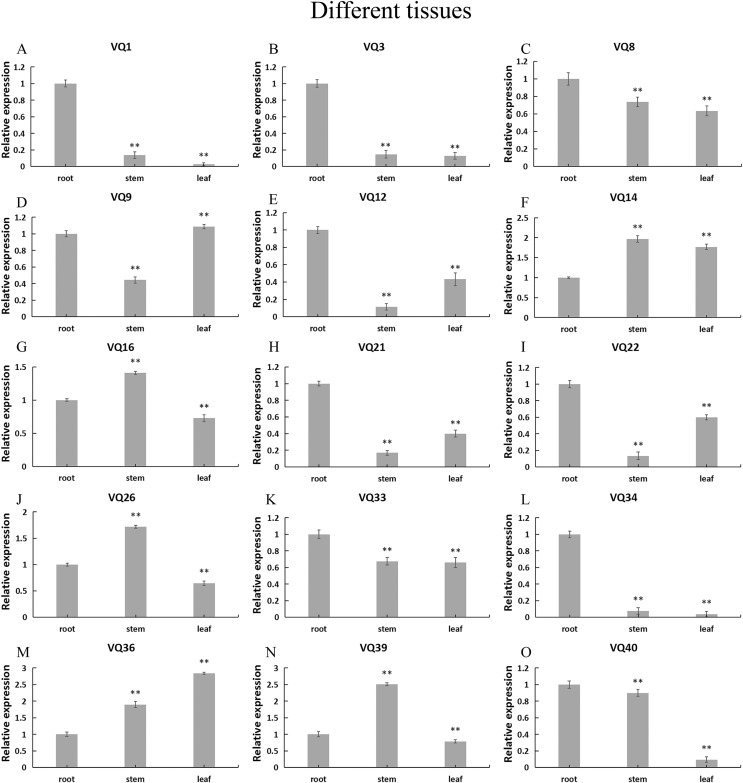
(A–O) Expression analysis of the *CpVQ* genes in different tissues of *C. pepo*. The surveyed tissues include root, stem, leaf. The 2^−ΔΔCt^ method was used to calculate the expression levels of target genes in different tissues.

In order to explore the possible role of *CpVQ* gene in the growth and development of *C. pepo*, we performed qRT-PCR expression analysis in three tissues of roots, stems and leaves. Expression patterns varied among the randomly selected 15 *CpVQ* genes (Fig. 6). Five *CpVQ* genes (*CaVQ1, 3, 12, 21* and *34*) were highly expressed in the root; two *CpVQ* genes (*CpVQ9* and *22*) were highly expressed in leaf and root; one *CpVQ* genes *CpVQ40* were highly expressed in stem and root; three *CpVQ* genes (*CpVQ16, 26* and *39*) were highly expressed in stem.

### Expression pattern of the *CpVQs* under powdery mildew stress

**Figure 7:**
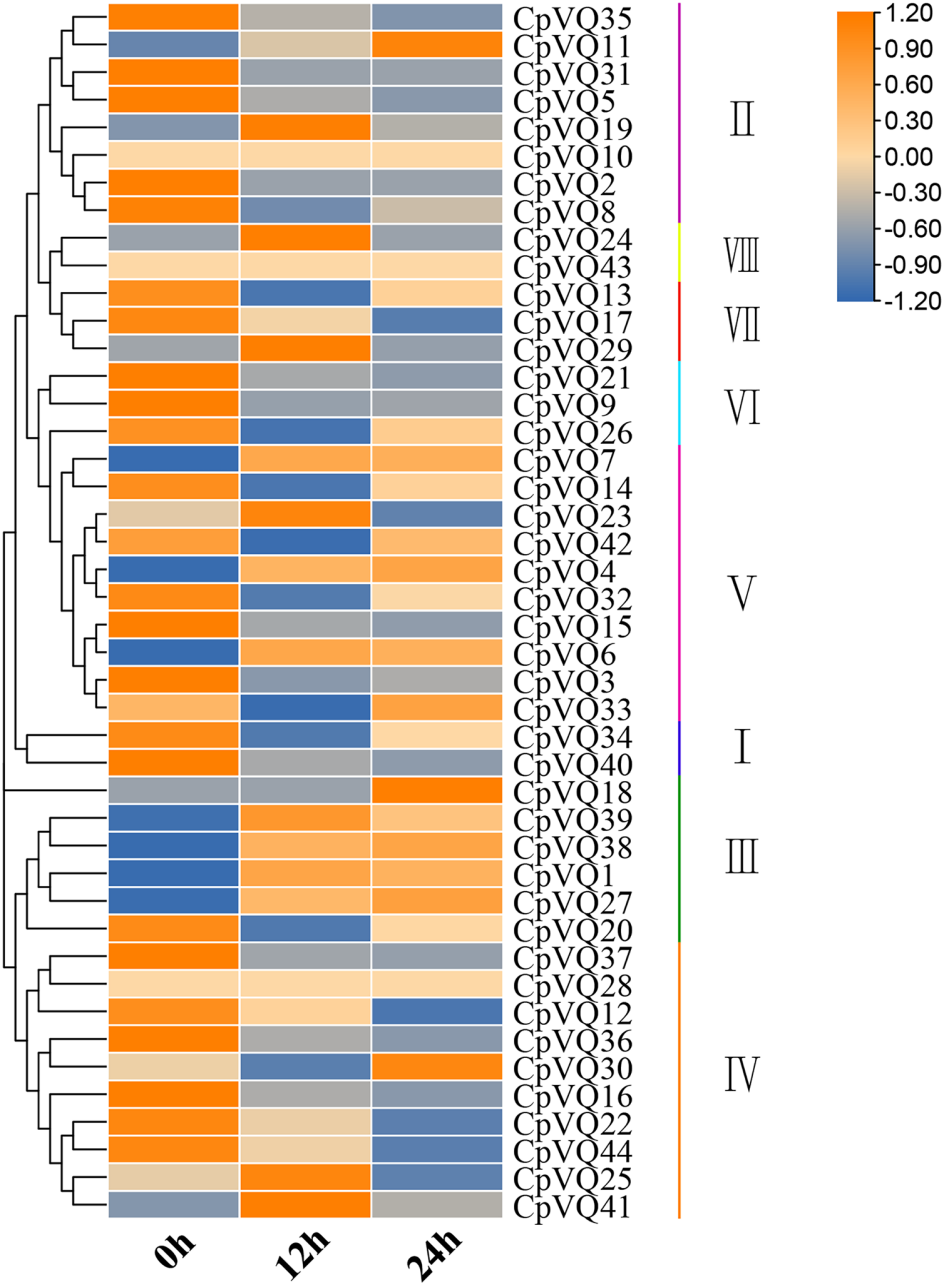
Expression profiles of *CpVQs* under powdery mildew stress based on the RNA-seq data. The gene expression values are square-root transformed fragments per kilo-bases per million mapped reads (FPKM). Different colors in map represent gene transcript abundance values as shown in the color bar.

**Figure 8:**
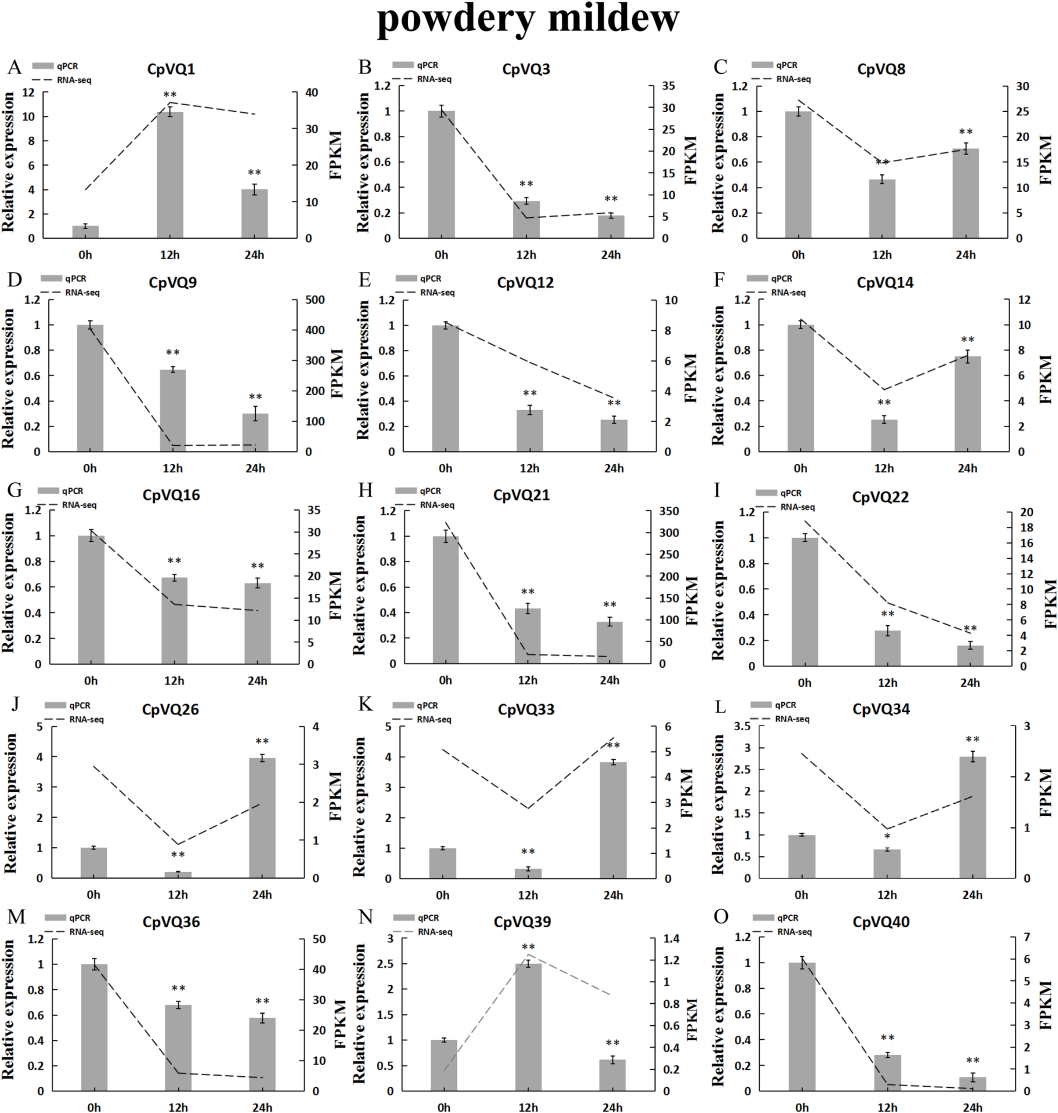
qRT-PCR validation of *VQ* genes in the response to powdery mildew treatment. Stress treatments and time course are described in “Materials & Methods”. (A–O) Different genes that were evaluated by qRT-PCR. Asterisks indicate statistically significant differences between the stressed samples and counterpart controls (*p < 0.05, **p < 0.01).

Several studies have shown that *VQs* play an important role in plant response to abiotic and biotic stress. Therefore, the expression profiles of *CpVQs* under powdery mildew stress were evaluated next based on RNA-seq data. Most of the *CpVQs* were found to be downregulated at early treatment timepoints, such as the members in group I and IV (Fig. 7). In addition, some members were downregulated and then upregulated. A total of 15 *CpVQs* from different groups were randomly selected for further validation by qRT-PCR (Fig. 8). Accordingly, most of the tested *CpVQs* were confirmed to be downregulated after powdery mildew infection, with the expression of these genes being correlated with the RNA-seq data at different treatment timepoints. Among these genes, the expression of two genes (*CpVQ1*, *CpVQ39*) significantly increased (more than 2-fold) at 12 h, whereas the expression of eight genes (*CpVQ3*, *CpVQ9*, *CpVQ12*, *CpVQ16*, *CpVQ21*, *CpVQ22*, *CpVQ36*, and *CpVQ40*) was significantly downregulated. In addition, three genes (*CpVQ26*, *CpVQ33*, and *CpVQ34*) had a high expression value at 24 h after powdery mildew stress.

### *CpVQs* gene expression following abiotic stress by qRT-PCR

To further investigate the role of *CpVQs* in abiotic stress responses, We randomly selected 15 *CpVQ* genes from eight groups, and made sure their responses to the drought-, cold-, salt-, and waterlogging-stress.

**Figure 9:**
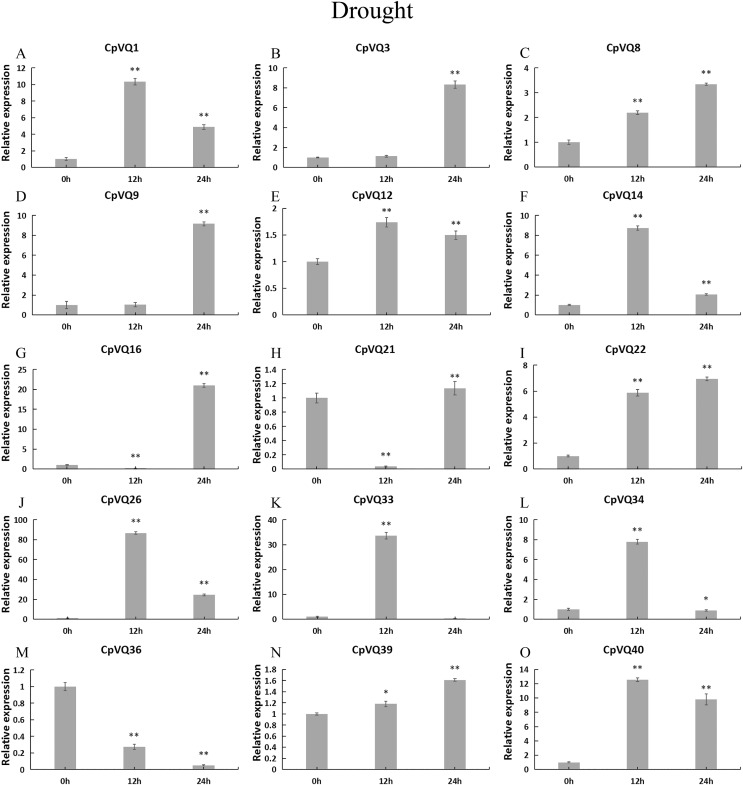
qRT-PCR validation of *VQ* genes in the response to drought treatment. Stress treatments and time course are described in “Materials & Methods”. (A–O) Different genes that were evaluated by qRT-PCR. Asterisks indicate statistically significant differences between the stressed samples and counterpart controls (*p < 0.05, **p < 0.01).

Under drought treatment (Fig. 9), all *CpVQs* were upregulated at different treatment timepoints. The expression of six genes (*CpVQ1*, *CpVQ14*, *CpVQ26*, *CpVQ33*, *CpVQ34*, and *CpVQ40*) significantly increased (more than 5-fold) at 12 h, whereas *CpVQ21* was significantly downregulated at 12 h but it then increased at 24 h. In addition, five genes (*CpVQ3*, *CpVQ8*, *CpVQ9*, *CpVQ16*, and *CpVQ39*) had a high expression value at 24 h after drought stress.

**Figure 10:**
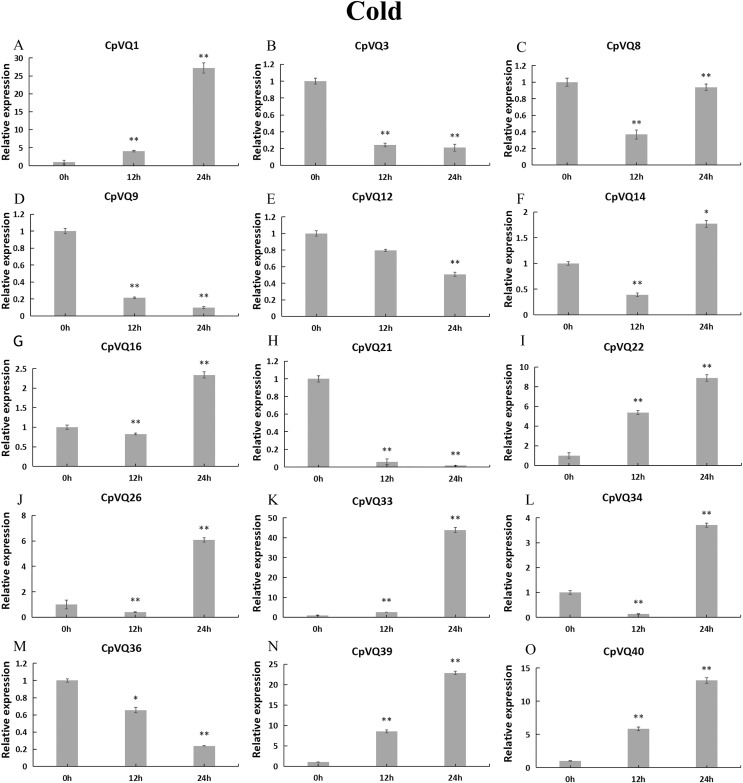
qRT-PCR validation of *VQ* genes in the response to cold treatment. Stress treatments and time course are described in “Materials & Methods”. (A–O) Different genes that were evaluated by qRT-PCR. Asterisks indicate statistically significant differences between the stressed samples and counterpart controls (*p < 0.05, **p < 0.01).

During cold stress (Fig. 10), the expression of eight *CpVQs* (*CpVQ1*, *CpVQ16*, *CpVQ22*, *CpVQ26*, *CpVQ33*, *CpVQ34*, *CpVQ39*, and *CpVQ40*) were significantly upregulated (more than 2-fold) at 24 h. In contrast, four genes (*CpVQ3*, *CpVQ9*, *CpVQ21* and *CpVQ36*) were downregulated at 12 h and their levels were further decreased at 24 h. Three members (*CpVQ8*, *CpVQ14*, and *CpVQ34*) were downregulated at early time points but their expression were significantly increased and peaked at 24 h.

**Figure 11:**
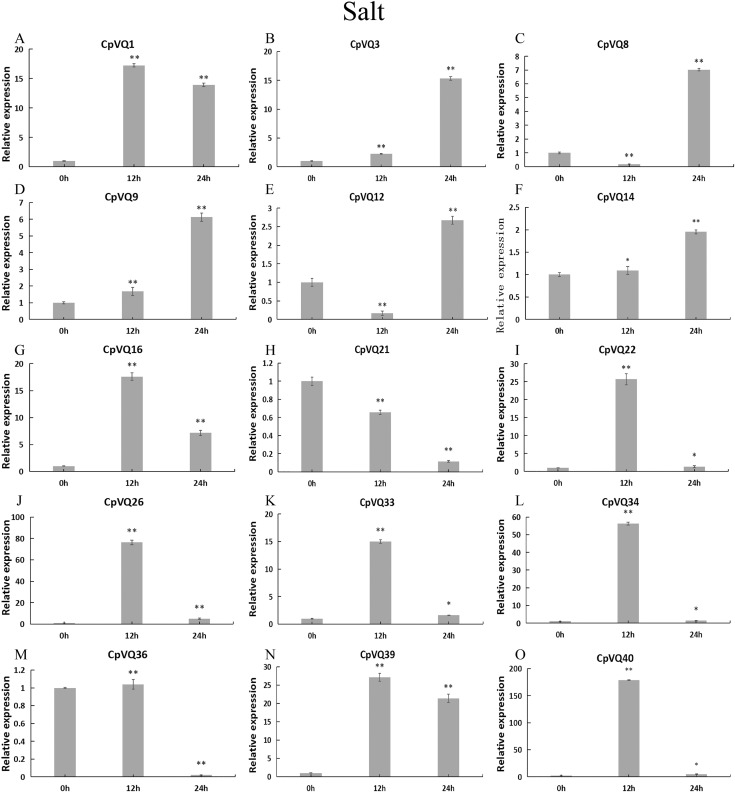
qRT-PCR validation of *VQ* genes in the response to salt treatment. Stress treatments and time course are described in “Materials & Methods”. (A–O) Different genes that were evaluated by qRT-PCR. Asterisks indicate statistically significant differences between the stressed samples and counterpart controls (*p < 0.05, **p < 0.01).

Upon salt stress (Fig. 11), six genes (*CpVQ16*, *CpVQ22*, *CpVQ26*, *CpVQ33*, *CpVQ34*, and *CpVQ40*) shared a similar expression trend, rapidly rising at early time points but with subsequently decreased expression. In contrast, *CpVQ8* and *CpVQ12* showed a trend of downregulation from 0 to 12 h, but then their levels were rapidly increased. In addition, the expression of *CpVQ21* showed a trend of downregulation from 0 to 24 h.

**Figure 12:**
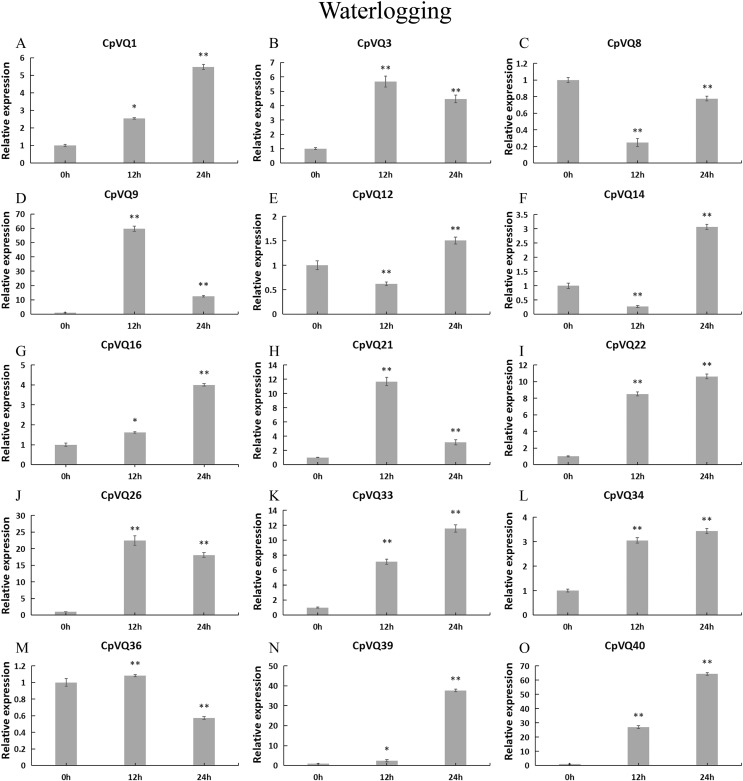
qRT-PCR validation of *VQ* genes in the response to waterlogging treatment. Stress treatments and time course are described in “Materials & Methods”. (A–O) Different genes that were evaluated by qRT-PCR. Asterisks indicate statistically significant differences between the stressed samples and counterpart controls (*p < 0.05, **p < 0.01).

For the case of waterlogging treatment (Fig. 12). The four genes (*CpVQ3*, *CpVQ9*, *CpVQ21* and *CpVQ26*) upregulated in the first 12 h and then downregulated in the next 24 h. In contrast, *CpVQ8*, *CpVQ12* and *CpVQ14* downregulated in the first 12 h and then upregulated in the next 24 h. Interesting, the expression of seven *CpVQs* (*CpVQ1*, *CpVQ16*, *CpVQ22*, *CpVQ33*, *CpVQ34*, *CpVQ39*, and *CpVQ40*) were upregulated from 0 to 24 h.

## Discussion

VQ has been proven to be the main transcriptional regulator in plants. Currently, the VQ family genes have been systematically analyzed in *Arabidopsis* (Cheng et al., 2012), rice (Kim et al., 2013a), maize (Song et al., 2016), tobacco (Liu et al., 2020) and cotton (Chen et al., 2020b), and responding to biotic and abiotic stresses (Jiang, Sevugan & Ramachandran, 2018; Perruc et al., 2004). However, current information on VQ characteristics in *C. pepo* is limited. Therefore, a comprehensive analysis of *VQ* genes in *C. pepo* and their expression patterns under various non-biological and powdery mildew treatments may pave the way for a better understanding of the mechanism of plant growth and development. This will also help to select candidate genes and lay the foundation for further in-depth study of the role of different regulatory networks in plant development and stress-related processes.

### Conservation of the *VQ* gene family of *C. pepo*

In higher eukaryotes, genes without introns are very common (Louhichi, Fourati & Rebaï, 2011; Liu et al., 2021). Herein, according to the gene structure, most *VQ* genes in *C. pepo* were also found to have no introns. Only 11 *VQs* had introns, among which *CpVQ14* contained four introns. This result is consistent with the lack of introns in 88.2% of *Arabidopsis*, 90% of Chinese Cabbage, and 92.3% of tomato (Cheng et al., 2012; Zhang et al., 2015; Ding et al., 2019). Subsequently, a phylogenetic tree was constructed based on the VQ proteins of *C. pepo* and *A. thaliana*. Through the analysis of the phylogenetic and structural features of the VQ domains, and these proteins were divided into eight clades (I–VIII) based on the nomenclature of the *A. thaliana VQs*. This analysis revealed that *VQs* with introns are located in different subfamilies, suggesting that these introns appear relatively independently. Comparative these plants (*Cicer arietinum* and *Medicago truncatula*, *A. thaliana* and lower plants, moss) indicate that most *VQ* genes have lost introns during the long evolutionary period. Taken together, *VQs* identified in *C. pepo* and in other species provide a certain reference value for the evolution of introns in plants. The average length of the *CpVQ* is 219.3 amino acids, a majority of the *C. pepo VQ* genes are intronless so they encode relatively small proteins with fewer than 300 amino acid residues, which is highly similar to those reported for *Nicotiana tabacum*, *Arabidopsis*, tomato, *O. sativa*, and other plants (Liu et al., 2020; Cheng et al., 2012; Ding et al., 2019; Kim et al., 2013a). Noteworthy, *CpVQ4*, *CpVQ14*, *CpVQ23*, and *CpVQ32* were all found to be close to 400 amino acids.

The genetic structure of the *VQ* gene and the conserved Motif not only appear in higher plants such as *Arabidopsis*, *O. sativa*, *Z. mays*, among others, but also in lower plants such as moss, which also implies their ancient origins in the evolutionary history and their important role in plant development (Jing & Lin, 2015; Song et al., 2016). Moreover, all 44 VQ proteins of *C. pepo* were found to harbor conserved VQ domains and contained the same type of Motifs, implying that in the same branch of the evolutionary tree, the closer the genes of VQ protein are, the more similar the gene structure is. Motif 1 was identified as the core Motif that composes the VQ domain, which was included in all pumpkin VQ proteins, thereby endowing the pumpkin VQ protein with specific biological functions. These results were in agreement with those reported for tomato and soybean species (Ding et al., 2019; Wang et al., 2019). In addition, VQ proteins with similar Motif composition were also found to be located in the same sub-branch of the evolutionary tree, as the Motifs composition between different branches was different.

### Expansion mechanism of the *C. pepo* VQ family

Genome replication events play an important role in expanding the size of the genome and diversifying gene functions, as replication events can result in genes with new functions (Rensing, 2014). In higher plants, tandem repeat events and chromosome fragment replication events are the main processes contributing to gene family expansion (Wang, Wang & Paterson, 2012b). Previous studies have shown that diverse WGD events lead to the different sizes of plant genomes (Adams & Wendel, 2005). In this study, 44 *VQ* genes were identified in *C. pepo*, which is a slightly high number of genes as compared with other species, such as *Arabidopsis* with 34 *VQ* members, or *O. sativa* and *Vitis vinifera* with 39 and 18 *VQs*, respectively (Cheng et al., 2012; Kim et al., 2013a; Wang et al., 2015a). Nevertheless, the number of VQ members in *C. pepo* was lower than initially predicted while considering its genome size of 261 Mb, as compared with the 125, 389, 486 Mb of *Arabidopsis*, *O. sativa*, and *V. vinifera*, respectively. Therefore, it can be concluded that there is no necessary connection between the size of the genome and the number of family members. Previous studies have shown that chromosome fragment replication is considered to be the main mechanism of *VQ* gene expansion (Tang et al., 2008); thus, the evolutionary process can explain the number of specific *VQ* genes in a species, not the size of the genome. The main process for the expansion of the *C. pepo* VQ family was found to be chromosome fragment duplication events, which is consistent with the results of previous studies. Among the 44 *CpVQs*, a total of 28 members participated in 21 chromosome fragment duplication events, accounting for 63.6%, and no tandem duplication event was identified. This is consistent with the expansion of the VQ family in *Brassica napus*, which is driven by chromosome fragment duplication, with tandem repeat events as the second driving force (Zou et al., 2021). Gene duplication can produce gene function redundancy, and these repeated genes can develop different gene expression patterns. We calculated the Ka, Ks and Ka/Ks ratios of all para-homologous gene pairs to explore the evolutionary constraints of repeated CpVQs. The Ka/Ks values of most gene pairs were less than 1.0, which indicated that these gene pairs had undergone purification selection pressure. In the present study, some synlinear genes showed different expression patterns, such as *CpVQ3* and *CpVQ33*, *CpVQ9* and *CpVQ21*, including under drought treatment, suggesting that these homologous genes in *C. pepo* may have different functions in regulation the normal growth and development of plants.

### Expression patterns of VQ members of *C. pepo*

Previous studies have shown that members of the VQ family play an important role in the entire plant development process and respond to various biotic and abiotic stresses. Molecular genetic evidence suggests that the plant VQ protein may be an important regulator of disease resistance and tolerance (Wang et al., 2015a). In this study, we analyzed the expression level of *CpVQ* gene in different tissues of *C. pepo* (Fig. 6). The results showed that most genes were differentially expressed in the tissues we analyzed. It indicates that the *CpVQ* gene may play an important role in the growth and development of these organs or tissues. In addition, based on transcriptome data, the expression *VQs* in *C. pepo* leaves under powdery mildew treatments at different timepoints was evaluated (as observed in Fig. 7). Most VQ members showed a significant decline in expression after 12 h of powdery mildew treatment, however, the expression patterns of the third group is different from the other groups. In the third group, except *CpVQ20*, all were significantly up-regulated upon powdery mildew infection, and three genes (*CpVQ18, 27* and *38*) had higher expression at 24 h, which may be because these genes responded late to powdery mildew stress. Studies have shown that *SIB1(AtVQ23)* and *SIB2(AtVQ16)* function as activators in plant defense against necrotrophic pathogens (Lai et al., 2011), so the *CpVQ* genes classified in the third group may have the same function. In contrast, after 24 h of powdery mildew treatment, the expression of most members in other groups decreased significantly. Similar results have been reported in grapevine that *VvVQ* genes are quickly responsive to powdery mildew stresses (Wang et al., 2015a). Therefore, the members of the VQ family in *C. pepo* may play a key role in the powdery mildew signaling pathway. Moreover, analysis of *VQs* expression under different abiotic stresses further demonstrated that the members of the VQ family are also significantly induced under different stresses. In this study, four *CpVQ* genes (*CpVQ3, 9, 16*, and *22*) significantly upregulated during drought stress (as observed in Fig. 9), which results are similar to the *OsVQ* genes that 22 *OsVQ* genes are upregulated under drought stress (Kim et al., 2013a). Under cold stress (as observed in Fig. 10), five *CaVQ* genes (*CaVQ1, 22, 33, 39* and *40*) were significantly induced. Similar results have been reported in Chinese cabbage that *BrVQ* genes are quickly responsive to cold stresses (Zhang et al., 2015). In addition, the *VQ* genes are also sensitive to salt changes. Two *CpVQ* genes (*CaVQ*3 and 9) were upregulated during salt treatment (as observed in Fig. 11). Similar changes occurred in Arabidopsis. *AtVQ9* and *AtVQ15* were significantly expressed under salt stress. Seven *CaVQ* genes (*CaVQ1, 16, 22, 33, 34, 39* and *40*) were upregulated at waterlogging treatment (as observed in Fig. 12). In summary, CpVQ members may be involved in regulating the response of plants to various abiotic stresses and powdery mildew stress, and their response mechanisms may be complex and diverse.

In summary, this study has systematically analyzed the evolutionary relationship, conserved structure, and expression patterns of the members of the VQ family of *C. pepo* at the whole genome level. The selection of candidate genes can provide reference for future investigations.

## Conclusions

In conclusion, this study provides the first comprehensive and systematic analysis of the 44 *VQ* genes identified in *C. pepo* genome. All *CpVQ* genes can be divided into eight groups (I–VIII), and were found to expand by chromosome fragment duplication events. RNA-seq analysis further showed that half of the *VQs* have significantly different expression patterns at different timepoints of powdery mildew infection. Therefore, the members of the VQ family in *C. pepo* may play a key role in the powdery mildew signaling pathway. Genetic analysis data further confirmed that the VQ family members respond to different abiotic stresses. Taken together, these findings provide a theoretical basis for further research on the functions of *CpVQs*.

## Supplemental Information

10.7717/peerj.12827/supp-1 Supplemental Information 1 Raw data.

10.7717/peerj.12827/supp-2 Supplemental Information 2 List of primers used in qRT-PCR.

## Additional Information and Declarations

### Data Availability

The following information was supplied regarding data availability: The raw measurements are available in the Supplemental File.
