# Supplementary material for: Genome-wide identification and expression analysis of the VQ gene family in Cucurbita pepo L
Source: PeerJ. 2022 Jan 21;10:e12827. doi: 10.7717/peerj.12827 (PMC8785662; doi:10.7717/peerj.12827)
Supplement: Supplemental Information 2 [file peerj-10-12827-s002.docx]

| **List of primers used in qRT-PCR** | | |
| --- | --- | --- |
| Gene name | Forward primer (5'-3') | Reverse primer(5'-3') |
| CpVQ1 | CACGGCGGATGCTGATCTGATG | ATAGAAAGAGCCAAGAAAGGGGTCATC |
| CpVQ3 | ACCACCTCCTTCCGATTTACCTACC | CCAGCCGAACTTCCGATGATAGC |
| CpVQ8 | TCGCCTCGCTGGAACAACTTAAC | TACTGCCGATGATGGTGGTGGAG |
| CpVQ9 | AGACGACGACGACTTCTTCAATTCC | GGTGTGATTCTTCTTGGAGGCTCTAG |
| CpVQ12 | CAAGCCGACCCAAACTCCTTCAG | ATGGTCTGTGGCGGGAGGTAAG |
| CpVQ14 | GGCGGCGAAGAAGATTACGACTC | GGGAGGAGGTGGGTTTGGTAGG |
| CpVQ16 | GCTCACTGGCTCATCGGAATCTTC | CTTGTGACGGCGGAGGAATAGAAC |
| CpVQ21 | GGACGCAAGATCACCAAGAGGAAG | GGACCATGTGGCGGAAGTTAGC |
| CpVQ22 | TCTGGGTTGGCGAATGCGAAATC | GTGTAGTGGTCGGTGATGGATGTAAG |
| CpVQ26 | CTATCAGCAGCAGCAGCGATCAG | CTGGGCTTGGAAATGGAGTTGTTATTG |
| CpVQ33 | CCGACGACCTTGCTCAATACAGAC | GATGAGGCGAAAGGCGGAGTTG |
| CpVQ34 | AACGGATGCCAAGAGCTTCAAGAC | CTCTCGCAGCACTCTTTGGAACTC |
| CpVQ36 | ATGGTTTGACAGGGATCGGAATTGG | GATCGCCTTCTCTTCCTCTTCACAAG |
| CpVQ39 | CCGCCGCCCAGGACTATGATAG | TCCGATGAGTGGAGGAGGAAGAATC |
| CpVQ40  CAC | AGTCCGTCGTTCAGACCCTCAC  GGACAAACAGAACCAACCATGA | GTTCTCATCCACTCGCCGCTTC  GGTTTCCTTTCCGTCACTGTAGA |
| action | CCTCTCAATCCCAAAGCTAACAG | CGGCCTGGATAGCAACATACA |
